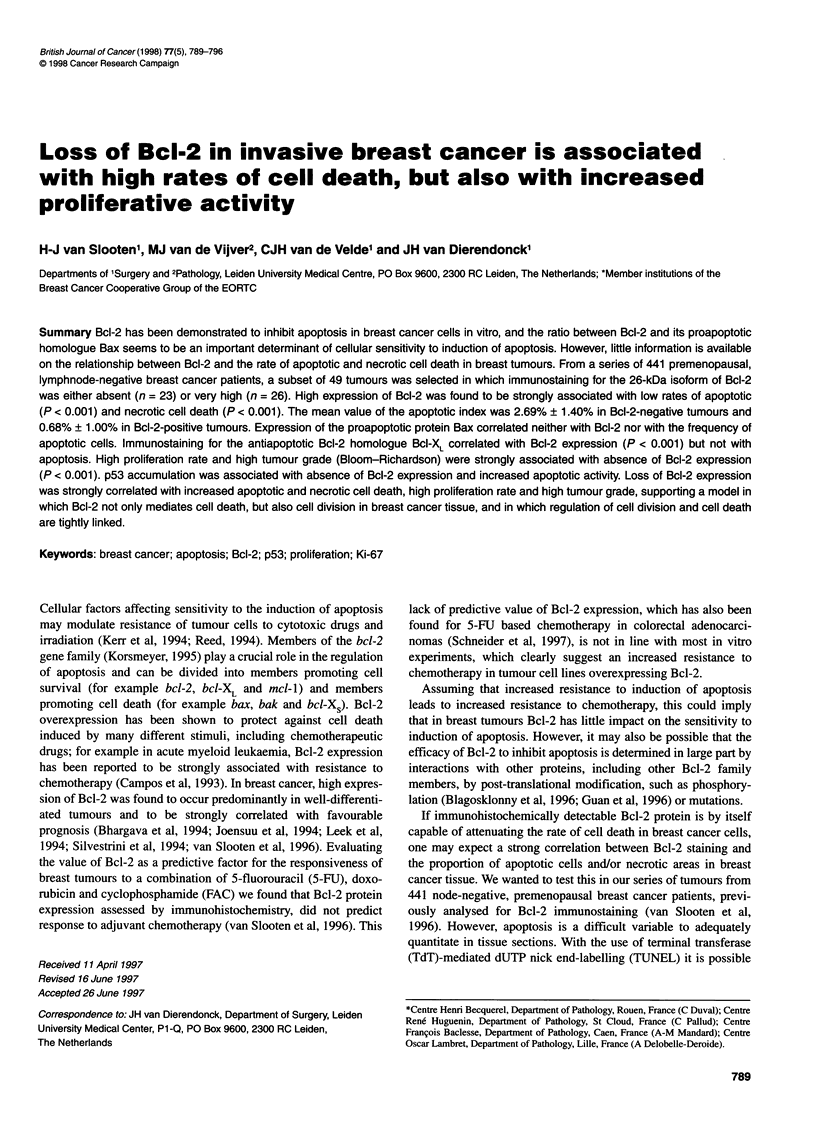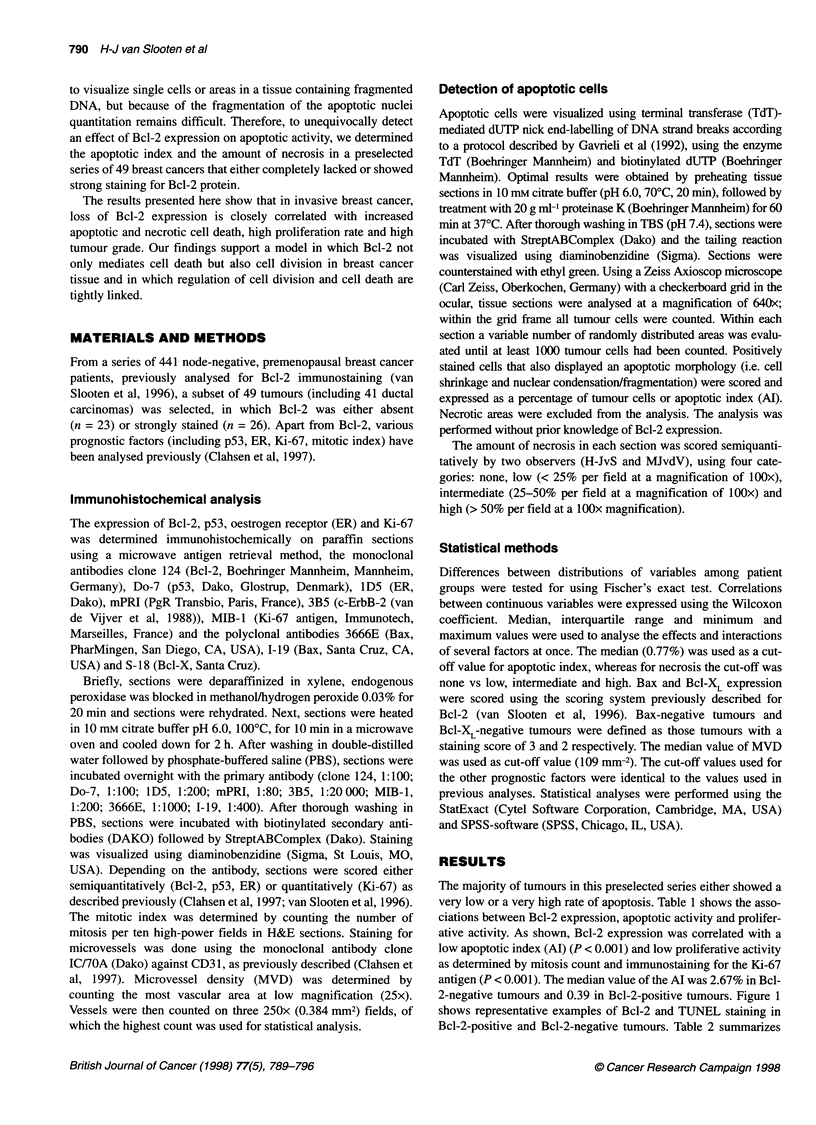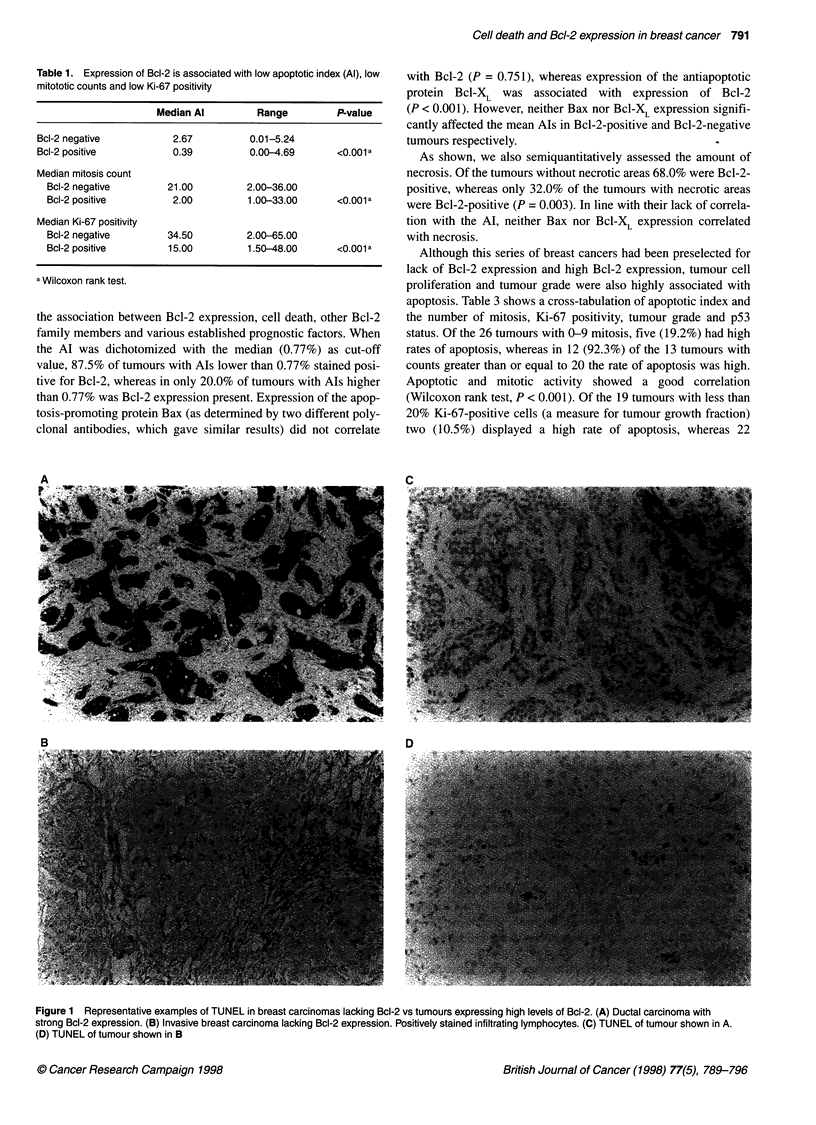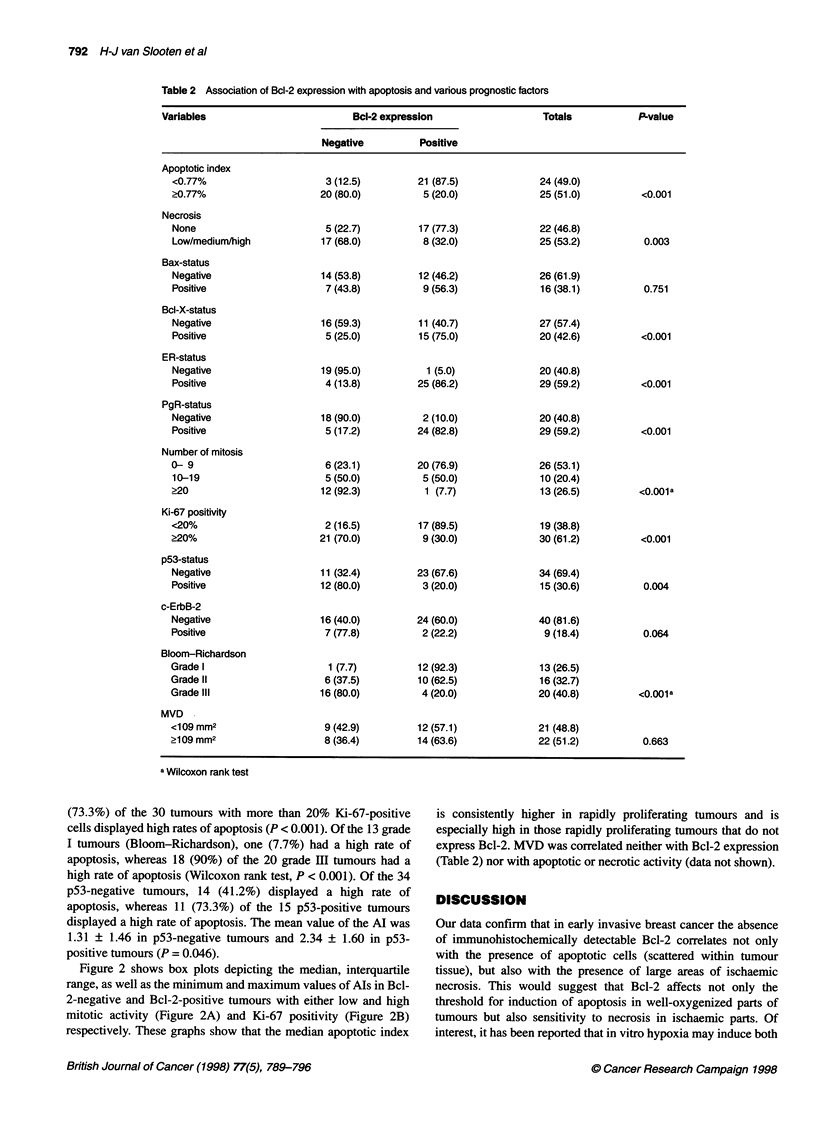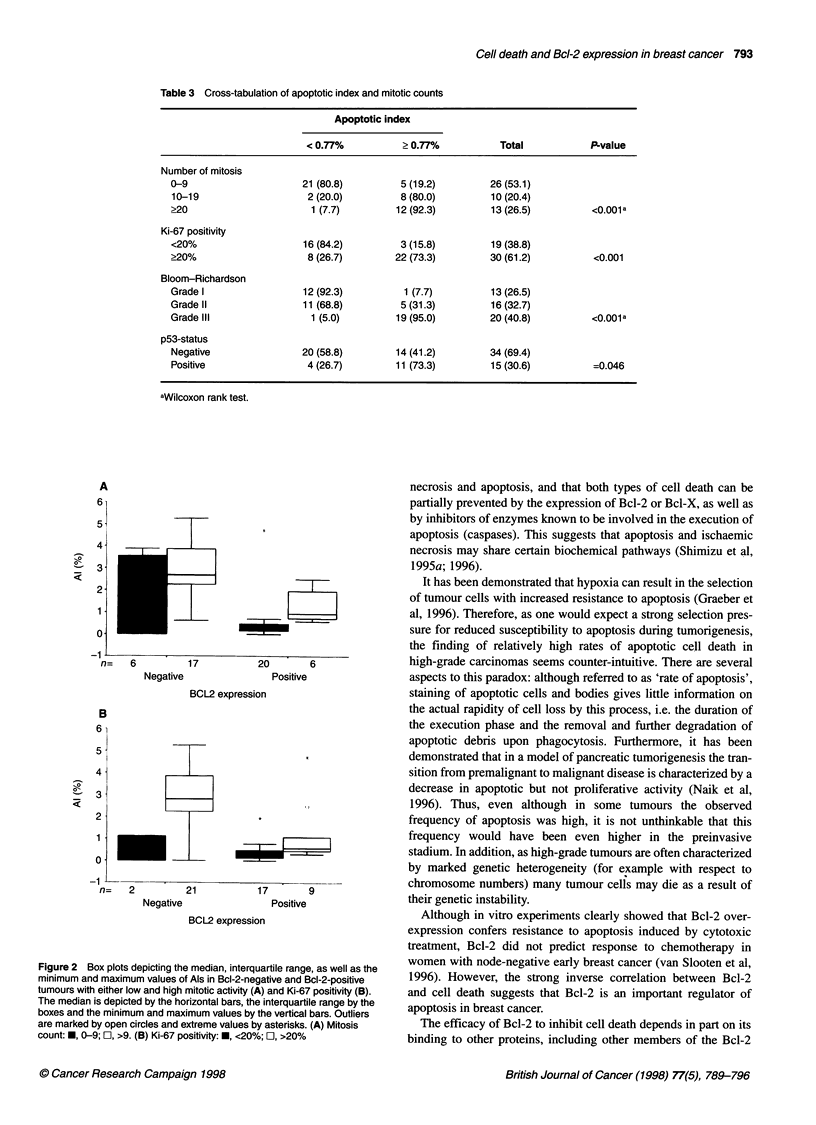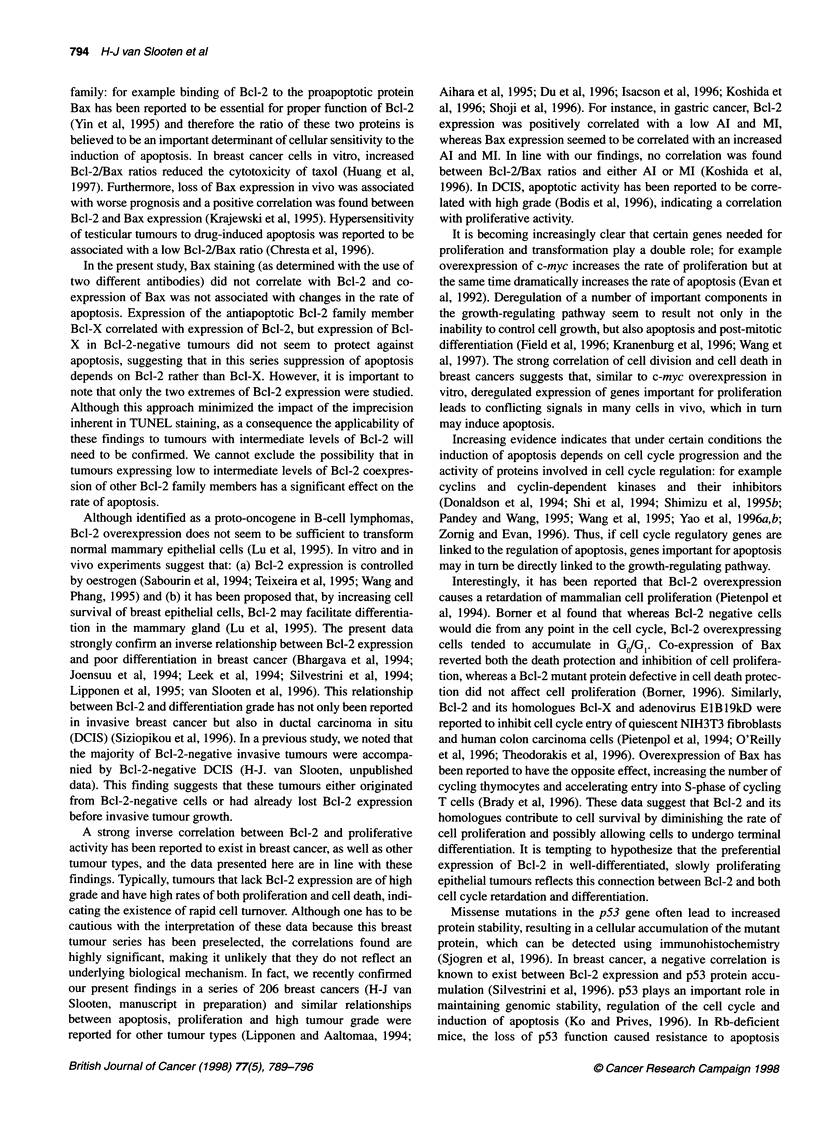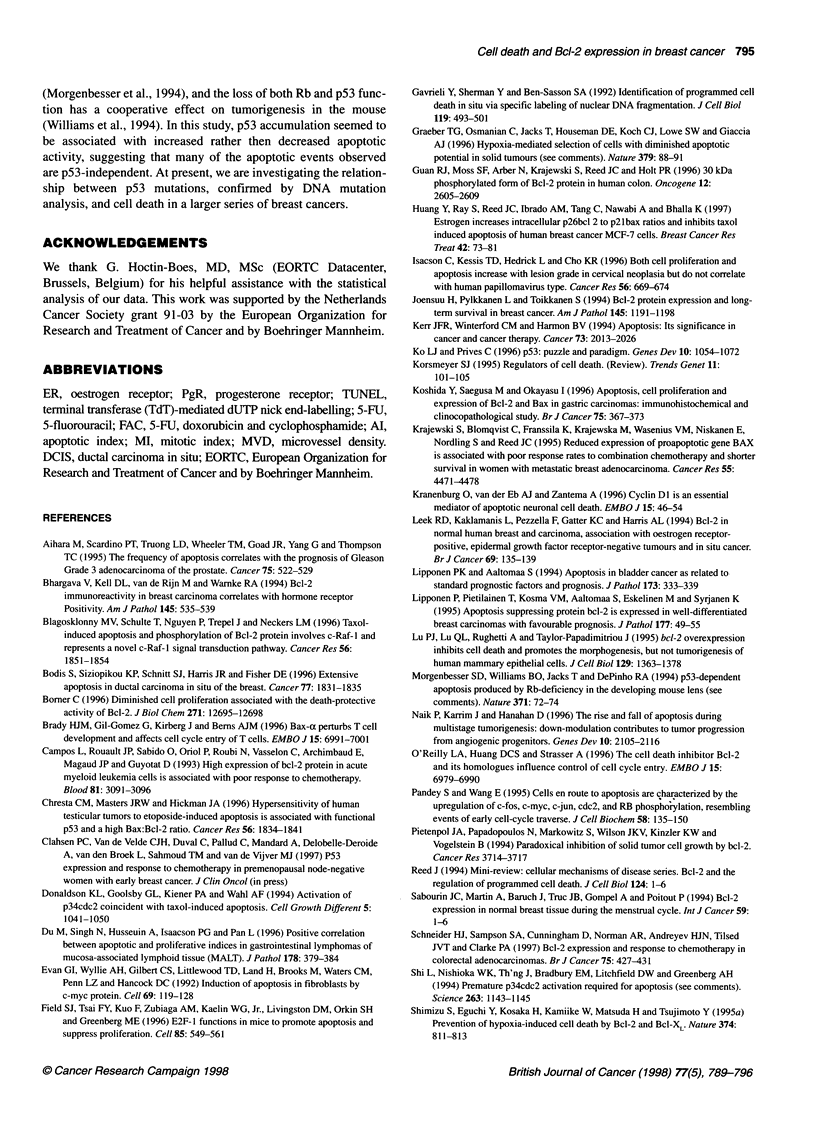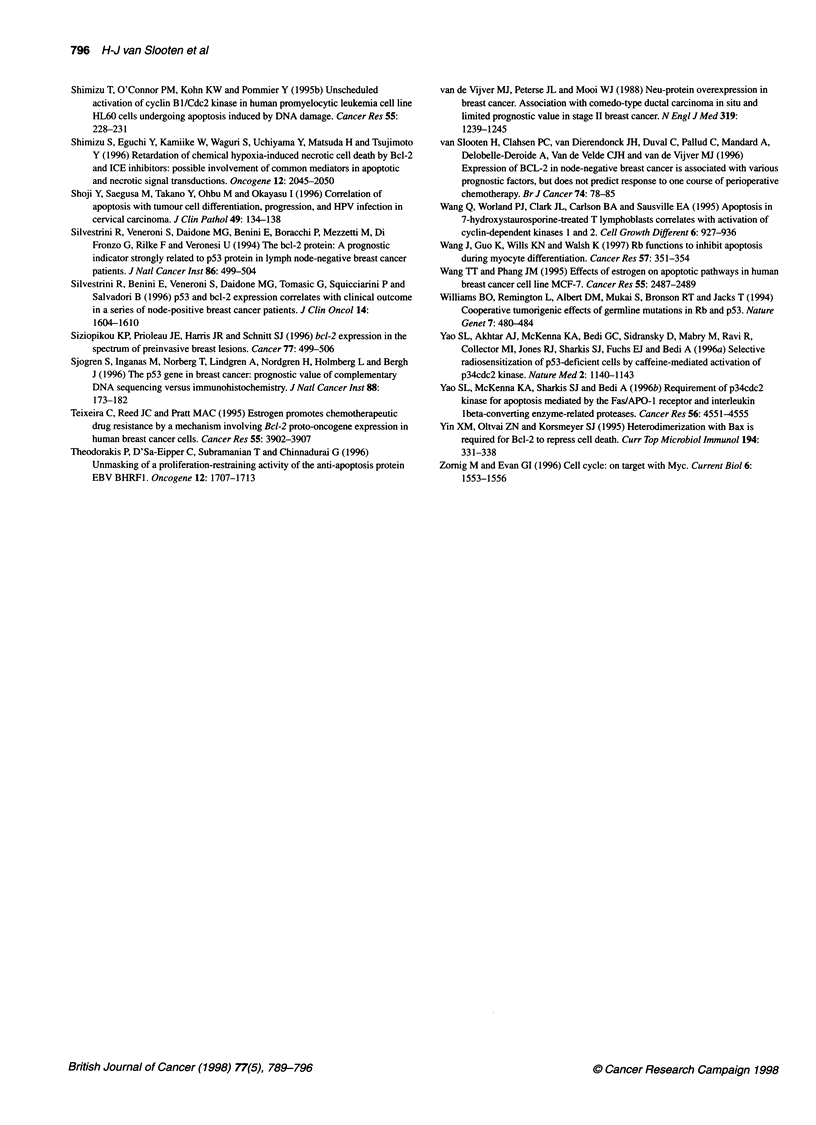# Loss of Bcl-2 in invasive breast cancer is associated with high rates of cell death, but also with increased proliferative activity.

**DOI:** 10.1038/bjc.1998.128

**Published:** 1998-03

**Authors:** H. J. van Slooten, M. J. van de Vijver, C. J. van de Velde, J. H. van Dierendonck

**Affiliations:** Department of Surgery, Leiden University Medical Centre, The Netherlands.

## Abstract

**Images:**


British Journal of Cancer (1998) 77(5), 789–796
© 1998 Cancer Research Campaign

### Loss of Bcl-2 in invasive breast cancer is associated with high rates of cell death, but also with increased proliferative activity

**H-J van Slooten[1], MJ van de Vijver[2], CJH van de Velde[1] and JH van Dierendonck[1]**

Departments of [1]Surgery and [2]Pathology, Leiden University Medical Centre, PO Box 9600, 2300 RC Leiden, The Netherlands; *Member institutions of the Breast Cancer Cooperative Group of the EORTC

**Summary** Bcl-2 has been demonstrated to inhibit apoptosis in breast cancer cells in vitro, and the ratio between Bcl-2 and its proapoptotic homologue Bax seems to be an important determinant of cellular sensitivity to induction of apoptosis. However, little information is available on the relationship between Bcl-2 and the rate of apoptotic and necrotic cell death in breast tumours. From a series of 441 premenopausal, lymphnode-negative breast cancer patients, a subset of 49 tumours was selected in which immunostaining for the 26-kDa isoform of Bcl-2 was either absent ($n = 23$) or very high ($n = 26$). High expression of Bcl-2 was found to be strongly associated with low rates of apoptotic ($P < 0.001$) and necrotic cell death ($P < 0.001$). The mean value of the apoptotic index was 2.69% ± 1.40% in Bcl-2-negative tumours and 0.68% ± 1.00% in Bcl-2-positive tumours. Expression of the proapoptotic protein Bax correlated neither with Bcl-2 nor with the frequency of apoptotic cells. Immunostaining for the antiapoptotic Bcl-2 homologue Bcl-$X_L$ correlated with Bcl-2 expression ($P < 0.001$) but not with apoptosis. High proliferation rate and high tumour grade (Bloom–Richardson) were strongly associated with absence of Bcl-2 expression ($P < 0.001$). p53 accumulation was associated with absence of Bcl-2 expression and increased apoptotic activity. Loss of Bcl-2 expression was strongly correlated with increased apoptotic and necrotic cell death, high proliferation rate and high tumour grade, supporting a model in which Bcl-2 not only mediates cell death, but also cell division in breast cancer tissue, and in which regulation of cell division and cell death are tightly linked.

**Keywords**: breast cancer; apoptosis; Bcl-2; p53; proliferation; Ki-67

Cellular factors affecting sensitivity to the induction of apoptosis may modulate resistance of tumour cells to cytotoxic drugs and irradiation (Kerr et al, 1994; Reed, 1994). Members of the *bcl-2* gene family (Korsmeyer, 1995) play a crucial role in the regulation of apoptosis and can be divided into members promoting cell survival (for example *bcl-2*, *bcl*-$X_L$ and *mcl*-1) and members promoting cell death (for example *bax*, *bak* and *bcl*-$X_S$). Bcl-2 overexpression has been shown to protect against cell death induced by many different stimuli, including chemotherapeutic drugs; for example in acute myeloid leukaemia, Bcl-2 expression has been reported to be strongly associated with resistance to chemotherapy (Campos et al, 1993). In breast cancer, high expression of Bcl-2 was found to occur predominantly in well-differentiated tumours and to be strongly correlated with favourable prognosis (Bhargava et al, 1994; Joensuu et al, 1994; Leek et al, 1994; Silvestrini et al, 1994; van Slooten et al, 1996). Evaluating the value of Bcl-2 as a predictive factor for the responsiveness of breast tumours to a combination of 5-fluorouracil (5-FU), doxorubicin and cyclophosphamide (FAC) we found that Bcl-2 protein expression assessed by immunohistochemistry, did not predict response to adjuvant chemotherapy (van Slooten et al, 1996). This lack of predictive value of Bcl-2 expression, which has also been found for 5-FU based chemotherapy in colorectal adenocarcinomas (Schneider et al, 1997), is not in line with most in vitro experiments, which clearly suggest an increased resistance to chemotherapy in tumour cell lines overexpressing Bcl-2.

Assuming that increased resistance to induction of apoptosis leads to increased resistance to chemotherapy, this could imply that in breast tumours Bcl-2 has little impact on the sensitivity to induction of apoptosis. However, it may also be possible that the efficacy of Bcl-2 to inhibit apoptosis is determined in large part by interactions with other proteins, including other Bcl-2 family members, by post-translational modification, such as phosphorylation (Blagosklonny et al, 1996; Guan et al, 1996) or mutations.

If immunohistochemically detectable Bcl-2 protein is by itself capable of attenuating the rate of cell death in breast cancer cells, one may expect a strong correlation between Bcl-2 staining and the proportion of apoptotic cells and/or necrotic areas in breast cancer tissue. We wanted to test this in our series of tumours from 441 node-negative, premenopausal breast cancer patients, previously analysed for Bcl-2 immunostaining (van Slooten et al, 1996). However, apoptosis is a difficult variable to adequately quantitate in tissue sections. With the use of terminal transferase (TdT)-mediated dUTP nick end-labelling (TUNEL) it is possible

*Received 11 April 1997*
*Revised 16 June 1997*
*Accepted 26 June 1997*

*Correspondence to:* JH van Dierendonck, Department of Surgery, Leiden University Medical Center, P1-Q, PO Box 9600, 2300 RC Leiden, The Netherlands

*Centre Henri Becquerel, Department of Pathology, Rouen, France (C Duval); Centre René Huguenin, Department of Pathology, St Cloud, France (C Pallud); Centre François Baclesse, Department of Pathology, Caen, France (A-M Mandard); Centre Oscar Lambret, Department of Pathology, Lille, France (A Delobelle-Deroide).

to visualize single cells or areas in a tissue containing fragmented DNA, but because of the fragmentation of the apoptotic nuclei quantitation remains difficult. Therefore, to unequivocally detect an effect of Bcl-2 expression on apoptotic activity, we determined the apoptotic index and the amount of necrosis in a preselected series of 49 breast cancers that either completely lacked or showed strong staining for Bcl-2 protein.

The results presented here show that in invasive breast cancer, loss of Bcl-2 expression is closely correlated with increased apoptotic and necrotic cell death, high proliferation rate and high tumour grade. Our findings support a model in which Bcl-2 not only mediates cell death but also cell division in breast cancer tissue and in which regulation of cell division and cell death are tightly linked.

### MATERIALS AND METHODS

From a series of 441 node-negative, premenopausal breast cancer patients, previously analysed for Bcl-2 immunostaining (van Slooten et al, 1996), a subset of 49 tumours (including 41 ductal carcinomas) was selected, in which Bcl-2 was either absent ($n$ = 23) or strongly stained ($n$ = 26). Apart from Bcl-2, various prognostic factors (including p53, ER, Ki-67, mitotic index) have been analysed previously (Clahsen et al, 1997).

### Immunohistochemical analysis

The expression of Bcl-2, p53, oestrogen receptor (ER) and Ki-67 was determined immunohistochemically on paraffin sections using a microwave antigen retrieval method, the monoclonal antibodies clone 124 (Bcl-2, Boehringer Mannheim, Mannheim, Germany), Do-7 (p53, Dako, Glostrup, Denmark), 1D5 (ER, Dako), mPRI (PgR Transbio, Paris, France), 3B5 (c-ErbB-2 (van de Vijver et al, 1988)), MIB-1 (Ki-67 antigen, Immunotech, Marseilles, France) and the polyclonal antibodies 3666E (Bax, PharMingen, San Diego, CA, USA), I-19 (Bax, Santa Cruz, CA, USA) and S-18 (Bcl-X, Santa Cruz).

Briefly, sections were deparaffinized in xylene, endogenous peroxidase was blocked in methanol/hydrogen peroxide 0.03% for 20 min and sections were rehydrated. Next, sections were heated in 10 mM citrate buffer pH 6.0, 100°C, for 10 min in a microwave oven and cooled down for 2 h. After washing in double-distilled water followed by phosphate-buffered saline (PBS), sections were incubated overnight with the primary antibody (clone 124, 1:100; Do-7, 1:100; 1D5, 1:200; mPRI, 1:80; 3B5, 1:20 000; MIB-1, 1:200; 3666E, 1:1000; I-19, 1:400). After thorough washing in PBS, sections were incubated with biotinylated secondary antibodies (DAKO) followed by StreptABComplex (Dako). Staining was visualized using diaminobenzidine (Sigma, St Louis, MO, USA). Depending on the antibody, sections were scored either semiquantitatively (Bcl-2, p53, ER) or quantitatively (Ki-67) as described previously (Clahsen et al, 1997; van Slooten et al, 1996). The mitotic index was determined by counting the number of mitosis per ten high-power fields in H&E sections. Staining for microvessels was done using the monoclonal antibody clone IC/70A (Dako) against CD31, as previously described (Clahsen et al, 1997). Microvessel density (MVD) was determined by counting the most vascular area at low magnification (25×). Vessels were then counted on three 250× (0.384 $mm^2$) fields, of which the highest count was used for statistical analysis.

### Detection of apoptotic cells

Apoptotic cells were visualized using terminal transferase (TdT)-mediated dUTP nick end-labelling of DNA strand breaks according to a protocol described by Gavrieli et al (1992), using the enzyme TdT (Boehringer Mannheim) and biotinylated dUTP (Boehringer Mannheim). Optimal results were obtained by preheating tissue sections in 10 mM citrate buffer (pH 6.0, 70°C, 20 min), followed by treatment with 20 g $ml^{-1}$ proteinase K (Boehringer Mannheim) for 60 min at 37°C. After thorough washing in TBS (pH 7.4), sections were incubated with StreptABComplex (Dako) and the tailing reaction was visualized using diaminobenzidine (Sigma). Sections were counterstained with ethyl green. Using a Zeiss Axioscop microscope (Carl Zeiss, Oberkochen, Germany) with a checkerboard grid in the ocular, tissue sections were analysed at a magnification of 640×; within the grid frame all tumour cells were counted. Within each section a variable number of randomly distributed areas was evaluated until at least 1000 tumour cells had been counted. Positively stained cells that also displayed an apoptotic morphology (i.e. cell shrinkage and nuclear condensation/fragmentation) were scored and expressed as a percentage of tumour cells or apoptotic index (AI). Necrotic areas were excluded from the analysis. The analysis was performed without prior knowledge of Bcl-2 expression.

The amount of necrosis in each section was scored semiquantitatively by two observers (H-JvS and MJvdV), using four categories: none, low (< 25% per field at a magnification of 100×), intermediate (25–50% per field at a magnification of 100×) and high (> 50% per field at a 100× magnification).

### Statistical methods

Differences between distributions of variables among patient groups were tested for using Fischer's exact test. Correlations between continuous variables were expressed using the Wilcoxon coefficient. Median, interquartile range and minimum and maximum values were used to analyse the effects and interactions of several factors at once. The median (0.77%) was used as a cut-off value for apoptotic index, whereas for necrosis the cut-off was none vs low, intermediate and high. Bax and Bcl-$X_L$ expression were scored using the scoring system previously described for Bcl-2 (van Slooten et al, 1996). Bax-negative tumours and Bcl-$X_L$-negative tumours were defined as those tumours with a staining score of 3 and 2 respectively. The median value of MVD was used as cut-off value (109 $mm^{-2}$). The cut-off values used for the other prognostic factors were identical to the values used in previous analyses. Statistical analyses were performed using the StatExact (Cytel Software Corporation, Cambridge, MA, USA) and SPSS-software (SPSS, Chicago, IL, USA).

### RESULTS

The majority of tumours in this preselected series either showed a very low or a very high rate of apoptosis. Table 1 shows the associations between Bcl-2 expression, apoptotic activity and proliferative activity. As shown, Bcl-2 expression was correlated with a low apoptotic index (AI) ($P < 0.001$) and low proliferative activity as determined by mitosis count and immunostaining for the Ki-67 antigen ($P < 0.001$). The median value of the AI was 2.67% in Bcl-2-negative tumours and 0.39 in Bcl-2-positive tumours. Figure 1 shows representative examples of Bcl-2 and TUNEL staining in Bcl-2-positive and Bcl-2-negative tumours. Table 2 summarizes

© Cancer Research Campaign 1998

**Table 1.** Expression of Bcl-2 is associated with low apoptotic index (AI), low mitototic counts and low Ki-67 positivity

| | Median AI | Range | *P*-value |
|---|---|---|---|
| Bcl-2 negative | 2.67 | 0.01–5.24 | |
| Bcl-2 positive | 0.39 | 0.00–4.69 | <0.001[a] |
| Median mitosis count | | | |
| Bcl-2 negative | 21.00 | 2.00–36.00 | |
| Bcl-2 positive | 2.00 | 1.00–33.00 | <0.001[a] |
| Median Ki-67 positivity | | | |
| Bcl-2 negative | 34.50 | 2.00–65.00 | |
| Bcl-2 positive | 15.00 | 1.50–48.00 | <0.001[a] |

[a] Wilcoxon rank test.

the association between Bcl-2 expression, cell death, other Bcl-2 family members and various established prognostic factors. When the AI was dichotomized with the median (0.77%) as cut-off value, 87.5% of tumours with AIs lower than 0.77% stained positive for Bcl-2, whereas in only 20.0% of tumours with AIs higher than 0.77% was Bcl-2 expression present. Expression of the apoptosis-promoting protein Bax (as determined by two different polyclonal antibodies, which gave similar results) did not correlate with Bcl-2 ($P = 0.751$), whereas expression of the antiapoptotic protein Bcl-$X_L$ was associated with expression of Bcl-2 ($P < 0.001$). However, neither Bax nor Bcl-$X_L$ expression significantly affected the mean AIs in Bcl-2-positive and Bcl-2-negative tumours respectively.

As shown, we also semiquantitatively assessed the amount of necrosis. Of the tumours without necrotic areas 68.0% were Bcl-2-positive, whereas only 32.0% of the tumours with necrotic areas were Bcl-2-positive ($P = 0.003$). In line with their lack of correlation with the AI, neither Bax nor Bcl-$X_L$ expression correlated with necrosis.

Although this series of breast cancers had been preselected for lack of Bcl-2 expression and high Bcl-2 expression, tumour cell proliferation and tumour grade were also highly associated with apoptosis. Table 3 shows a cross-tabulation of apoptotic index and the number of mitosis, Ki-67 positivity, tumour grade and p53 status. Of the 26 tumours with 0–9 mitosis, five (19.2%) had high rates of apoptosis, whereas in 12 (92.3%) of the 13 tumours with counts greater than or equal to 20 the rate of apoptosis was high. Apoptotic and mitotic activity showed a good correlation (Wilcoxon rank test, $P < 0.001$). Of the 19 tumours with less than 20% Ki-67-positive cells (a measure for tumour growth fraction) two (10.5%) displayed a high rate of apoptosis, whereas 22

**Figure 1** Representative examples of TUNEL in breast carcinomas lacking Bcl-2 vs tumours expressing high levels of Bcl-2. (**A**) Ductal carcinoma with strong Bcl-2 expression. (**B**) Invasive breast carcinoma lacking Bcl-2 expression. Positively stained infiltrating lymphocytes. (**C**) TUNEL of tumour shown in A. (**D**) TUNEL of tumour shown in **B**

© Cancer Research Campaign 1998 

**Table 2** Association of Bcl-2 expression with apoptosis and various prognostic factors

| Variables | Bcl-2 expression | | Totals | P-value |
|---|---|---|---|---|
| | Negative | Positive | | |
| Apoptotic index | | | | |
| <0.77% | 3 (12.5) | 21 (87.5) | 24 (49.0) | |
| ≥0.77% | 20 (80.0) | 5 (20.0) | 25 (51.0) | <0.001 |
| Necrosis | | | | |
| None | 5 (22.7) | 17 (77.3) | 22 (46.8) | |
| Low/medium/high | 17 (68.0) | 8 (32.0) | 25 (53.2) | 0.003 |
| Bax-status | | | | |
| Negative | 14 (53.8) | 12 (46.2) | 26 (61.9) | |
| Positive | 7 (43.8) | 9 (56.3) | 16 (38.1) | 0.751 |
| Bcl-X-status | | | | |
| Negative | 16 (59.3) | 11 (40.7) | 27 (57.4) | |
| Positive | 5 (25.0) | 15 (75.0) | 20 (42.6) | <0.001 |
| ER-status | | | | |
| Negative | 19 (95.0) | 1 (5.0) | 20 (40.8) | |
| Positive | 4 (13.8) | 25 (86.2) | 29 (59.2) | <0.001 |
| PgR-status | | | | |
| Negative | 18 (90.0) | 2 (10.0) | 20 (40.8) | |
| Positive | 5 (17.2) | 24 (82.8) | 29 (59.2) | <0.001 |
| Number of mitosis | | | | |
| 0– 9 | 6 (23.1) | 20 (76.9) | 26 (53.1) | |
| 10–19 | 5 (50.0) | 5 (50.0) | 10 (20.4) | |
| ≥20 | 12 (92.3) | 1 (7.7) | 13 (26.5) | <0.001[a] |
| Ki-67 positivity | | | | |
| <20% | 2 (16.5) | 17 (89.5) | 19 (38.8) | |
| ≥20% | 21 (70.0) | 9 (30.0) | 30 (61.2) | <0.001 |
| p53-status | | | | |
| Negative | 11 (32.4) | 23 (67.6) | 34 (69.4) | |
| Positive | 12 (80.0) | 3 (20.0) | 15 (30.6) | 0.004 |
| c-ErbB-2 | | | | |
| Negative | 16 (40.0) | 24 (60.0) | 40 (81.6) | |
| Positive | 7 (77.8) | 2 (22.2) | 9 (18.4) | 0.064 |
| Bloom–Richardson | | | | |
| Grade I | 1 (7.7) | 12 (92.3) | 13 (26.5) | |
| Grade II | 6 (37.5) | 10 (62.5) | 16 (32.7) | |
| Grade III | 16 (80.0) | 4 (20.0) | 20 (40.8) | <0.001[a] |
| MVD | | | | |
| <109 mm$^2$ | 9 (42.9) | 12 (57.1) | 21 (48.8) | |
| ≥109 mm$^2$ | 8 (36.4) | 14 (63.6) | 22 (51.2) | 0.663 |

[a] Wilcoxon rank test

(73.3%) of the 30 tumours with more than 20% Ki-67-positive cells displayed high rates of apoptosis ($P < 0.001$). Of the 13 grade I tumours (Bloom–Richardson), one (7.7%) had a high rate of apoptosis, whereas 18 (90%) of the 20 grade III tumours had a high rate of apoptosis (Wilcoxon rank test, $P < 0.001$). Of the 34 p53-negative tumours, 14 (41.2%) displayed a high rate of apoptosis, whereas 11 (73.3%) of the 15 p53-positive tumours displayed a high rate of apoptosis. The mean value of the AI was 1.31 ± 1.46 in p53-negative tumours and 2.34 ± 1.60 in p53-positive tumours ($P = 0.046$).

Figure 2 shows box plots depicting the median, interquartile range, as well as the minimum and maximum values of AIs in Bcl-2-negative and Bcl-2-positive tumours with either low and high mitotic activity (Figure 2A) and Ki-67 positivity (Figure 2B) respectively. These graphs show that the median apoptotic index is consistently higher in rapidly proliferating tumours and is especially high in those rapidly proliferating tumours that do not express Bcl-2. MVD was correlated neither with Bcl-2 expression (Table 2) nor with apoptotic or necrotic activity (data not shown).

### DISCUSSION

Our data confirm that in early invasive breast cancer the absence of immunohistochemically detectable Bcl-2 correlates not only with the presence of apoptotic cells (scattered within tumour tissue), but also with the presence of large areas of ischaemic necrosis. This would suggest that Bcl-2 affects not only the threshold for induction of apoptosis in well-oxygenized parts of tumours but also sensitivity to necrosis in ischaemic parts. Of interest, it has been reported that in vitro hypoxia may induce both

© Cancer Research Campaign 1998

**Table 3** Cross-tabulation of apoptotic index and mitotic counts

| | Apoptotic index | | | |
|---|---|---|---|---|
| | < 0.77% | ≥ 0.77% | Total | *P*-value |
| Number of mitosis | | | | |
| 0–9 | 21 (80.8) | 5 (19.2) | 26 (53.1) | |
| 10–19 | 2 (20.0) | 8 (80.0) | 10 (20.4) | |
| ≥20 | 1 (7.7) | 12 (92.3) | 13 (26.5) | <0.001[a] |
| Ki-67 positivity | | | | |
| <20% | 16 (84.2) | 3 (15.8) | 19 (38.8) | |
| ≥20% | 8 (26.7) | 22 (73.3) | 30 (61.2) | <0.001 |
| Bloom–Richardson | | | | |
| Grade I | 12 (92.3) | 1 (7.7) | 13 (26.5) | |
| Grade II | 11 (68.8) | 5 (31.3) | 16 (32.7) | |
| Grade III | 1 (5.0) | 19 (95.0) | 20 (40.8) | <0.001[a] |
| p53-status | | | | |
| Negative | 20 (58.8) | 14 (41.2) | 34 (69.4) | |
| Positive | 4 (26.7) | 11 (73.3) | 15 (30.6) | =0.046 |

[a]Wilcoxon rank test.

**Figure 2** Box plots depicting the median, interquartile range, as well as the minimum and maximum values of AIs in Bcl-2-negative and Bcl-2-positive tumours with either low and high mitotic activity (**A**) and Ki-67 positivity (**B**). The median is depicted by the horizontal bars, the interquartile range by the boxes and the minimum and maximum values by the vertical bars. Outliers are marked by open circles and extreme values by asterisks. (**A**) Mitosis count: ■, 0–9; □, >9. (**B**) Ki-67 positivity: ■, <20%; □, >20%

necrosis and apoptosis, and that both types of cell death can be partially prevented by the expression of Bcl-2 or Bcl-X, as well as by inhibitors of enzymes known to be involved in the execution of apoptosis (caspases). This suggests that apoptosis and ischaemic necrosis may share certain biochemical pathways (Shimizu et al, 1995*a*; 1996).

It has been demonstrated that hypoxia can result in the selection of tumour cells with increased resistance to apoptosis (Graeber et al, 1996). Therefore, as one would expect a strong selection pressure for reduced susceptibility to apoptosis during tumorigenesis, the finding of relatively high rates of apoptotic cell death in high-grade carcinomas seems counter-intuitive. There are several aspects to this paradox: although referred to as 'rate of apoptosis', staining of apoptotic cells and bodies gives little information on the actual rapidity of cell loss by this process, i.e. the duration of the execution phase and the removal and further degradation of apoptotic debris upon phagocytosis. Furthermore, it has been demonstrated that in a model of pancreatic tumorigenesis the transition from premalignant to malignant disease is characterized by a decrease in apoptotic but not proliferative activity (Naik et al, 1996). Thus, even although in some tumours the observed frequency of apoptosis was high, it is not unthinkable that this frequency would have been even higher in the preinvasive stadium. In addition, as high-grade tumours are often characterized by marked genetic heterogeneity (for example with respect to chromosome numbers) many tumour cells may die as a result of their genetic instability.

Although in vitro experiments clearly showed that Bcl-2 overexpression confers resistance to apoptosis induced by cytotoxic treatment, Bcl-2 did not predict response to chemotherapy in women with node-negative early breast cancer (van Slooten et al, 1996). However, the strong inverse correlation between Bcl-2 and cell death suggests that Bcl-2 is an important regulator of apoptosis in breast cancer.

The efficacy of Bcl-2 to inhibit cell death depends in part on its binding to other proteins, including other members of the Bcl-2

family: for example binding of Bcl-2 to the proapoptotic protein Bax has been reported to be essential for proper function of Bcl-2 (Yin et al, 1995) and therefore the ratio of these two proteins is believed to be an important determinant of cellular sensitivity to the induction of apoptosis. In breast cancer cells in vitro, increased Bcl-2/Bax ratios reduced the cytotoxicity of taxol (Huang et al, 1997). Furthermore, loss of Bax expression in vivo was associated with worse prognosis and a positive correlation was found between Bcl-2 and Bax expression (Krajewski et al, 1995). Hypersensitivity of testicular tumours to drug-induced apoptosis was reported to be associated with a low Bcl-2/Bax ratio (Chresta et al, 1996).

In the present study, Bax staining (as determined with the use of two different antibodies) did not correlate with Bcl-2 and co-expression of Bax was not associated with changes in the rate of apoptosis. Expression of the antiapoptotic Bcl-2 family member Bcl-X correlated with expression of Bcl-2, but expression of Bcl-X in Bcl-2-negative tumours did not seem to protect against apoptosis, suggesting that in this series suppression of apoptosis depends on Bcl-2 rather than Bcl-X. However, it is important to note that only the two extremes of Bcl-2 expression were studied. Although this approach minimized the impact of the imprecision inherent in TUNEL staining, as a consequence the applicability of these findings to tumours with intermediate levels of Bcl-2 will need to be confirmed. We cannot exclude the possibility that in tumours expressing low to intermediate levels of Bcl-2 coexpression of other Bcl-2 family members has a significant effect on the rate of apoptosis.

Although identified as a proto-oncogene in B-cell lymphomas, Bcl-2 overexpression does not seem to be sufficient to transform normal mammary epithelial cells (Lu et al, 1995). In vitro and in vivo experiments suggest that: (a) Bcl-2 expression is controlled by oestrogen (Sabourin et al, 1994; Teixeira et al, 1995; Wang and Phang, 1995) and (b) it has been proposed that, by increasing cell survival of breast epithelial cells, Bcl-2 may facilitate differentiation in the mammary gland (Lu et al, 1995). The present data strongly confirm an inverse relationship between Bcl-2 expression and poor differentiation in breast cancer (Bhargava et al, 1994; Joensuu et al, 1994; Leek et al, 1994; Silvestrini et al, 1994; Lipponen et al, 1995; van Slooten et al, 1996). This relationship between Bcl-2 and differentiation grade has not only been reported in invasive breast cancer but also in ductal carcinoma in situ (DCIS) (Siziopikou et al, 1996). In a previous study, we noted that the majority of Bcl-2-negative invasive tumours were accompanied by Bcl-2-negative DCIS (H-J. van Slooten, unpublished data). This finding suggests that these tumours either originated from Bcl-2-negative cells or had already lost Bcl-2 expression before invasive tumour growth.

A strong inverse correlation between Bcl-2 and proliferative activity has been reported to exist in breast cancer, as well as other tumour types, and the data presented here are in line with these findings. Typically, tumours that lack Bcl-2 expression are of high grade and have high rates of both proliferation and cell death, indicating the existence of rapid cell turnover. Although one has to be cautious with the interpretation of these data because this breast tumour series has been preselected, the correlations found are highly significant, making it unlikely that they do not reflect an underlying biological mechanism. In fact, we recently confirmed our present findings in a series of 206 breast cancers (H-J van Slooten, manuscript in preparation) and similar relationships between apoptosis, proliferation and high tumour grade were reported for other tumour types (Lipponen and Aaltomaa, 1994; Aihara et al, 1995; Du et al, 1996; Isacson et al, 1996; Koshida et al, 1996; Shoji et al, 1996). For instance, in gastric cancer, Bcl-2 expression was positively correlated with a low AI and MI, whereas Bax expression seemed to be correlated with an increased AI and MI. In line with our findings, no correlation was found between Bcl-2/Bax ratios and either AI or MI (Koshida et al, 1996). In DCIS, apoptotic activity has been reported to be correlated with high grade (Bodis et al, 1996), indicating a correlation with proliferative activity.

It is becoming increasingly clear that certain genes needed for proliferation and transformation play a double role; for example overexpression of c-*myc* increases the rate of proliferation but at the same time dramatically increases the rate of apoptosis (Evan et al, 1992). Deregulation of a number of important components in the growth-regulating pathway seem to result not only in the inability to control cell growth, but also apoptosis and post-mitotic differentiation (Field et al, 1996; Kranenburg et al, 1996; Wang et al, 1997). The strong correlation of cell division and cell death in breast cancers suggests that, similar to c-*myc* overexpression in vitro, deregulated expression of genes important for proliferation leads to conflicting signals in many cells in vivo, which in turn may induce apoptosis.

Increasing evidence indicates that under certain conditions the induction of apoptosis depends on cell cycle progression and the activity of proteins involved in cell cycle regulation: for example cyclins and cyclin-dependent kinases and their inhibitors (Donaldson et al, 1994; Shi et al, 1994; Shimizu et al, 1995*b*; Pandey and Wang, 1995; Wang et al, 1995; Yao et al, 1996*a,b*; Zornig and Evan, 1996). Thus, if cell cycle regulatory genes are linked to the regulation of apoptosis, genes important for apoptosis may in turn be directly linked to the growth-regulating pathway.

Interestingly, it has been reported that Bcl-2 overexpression causes a retardation of mammalian cell proliferation (Pietenpol et al, 1994). Borner et al found that whereas Bcl-2 negative cells would die from any point in the cell cycle, Bcl-2 overexpressing cells tended to accumulate in $G_0/G_1$. Co-expression of Bax reverted both the death protection and inhibition of cell proliferation, whereas a Bcl-2 mutant protein defective in cell death protection did not affect cell proliferation (Borner, 1996). Similarly, Bcl-2 and its homologues Bcl-X and adenovirus E1B19kD were reported to inhibit cell cycle entry of quiescent NIH3T3 fibroblasts and human colon carcinoma cells (Pietenpol et al, 1994; O'Reilly et al, 1996; Theodorakis et al, 1996). Overexpression of Bax has been reported to have the opposite effect, increasing the number of cycling thymocytes and accelerating entry into S-phase of cycling T cells (Brady et al, 1996). These data suggest that Bcl-2 and its homologues contribute to cell survival by diminishing the rate of cell proliferation and possibly allowing cells to undergo terminal differentiation. It is tempting to hypothesize that the preferential expression of Bcl-2 in well-differentiated, slowly proliferating epithelial tumours reflects this connection between Bcl-2 and both cell cycle retardation and differentiation.

Missense mutations in the *p53* gene often lead to increased protein stability, resulting in a cellular accumulation of the mutant protein, which can be detected using immunohistochemistry (Sjogren et al, 1996). In breast cancer, a negative correlation is known to exist between Bcl-2 expression and p53 protein accumulation (Silvestrini et al, 1996). p53 plays an important role in maintaining genomic stability, regulation of the cell cycle and induction of apoptosis (Ko and Prives, 1996). In Rb-deficient mice, the loss of p53 function caused resistance to apoptosis

 © Cancer Research Campaign 1998

(Morgenbesser et al., 1994), and the loss of both Rb and p53 function has a cooperative effect on tumorigenesis in the mouse (Williams et al., 1994). In this study, p53 accumulation seemed to be associated with increased rather then decreased apoptotic activity, suggesting that many of the apoptotic events observed are p53-independent. At present, we are investigating the relationship between p53 mutations, confirmed by DNA mutation analysis, and cell death in a larger series of breast cancers.

### ACKNOWLEDGEMENTS

We thank G. Hoctin-Boes, MD, MSc (EORTC Datacenter, Brussels, Belgium) for his helpful assistance with the statistical analysis of our data. This work was supported by the Netherlands Cancer Society grant 91-03 by the European Organization for Research and Treatment of Cancer and by Boehringer Mannheim.

### ABBREVIATIONS

ER, oestrogen receptor; PgR, progesterone receptor; TUNEL, terminal transferase (TdT)-mediated dUTP nick end-labelling; 5-FU, 5-fluorouracil; FAC, 5-FU, doxorubicin and cyclophosphamide; AI, apoptotic index; MI, mitotic index; MVD, microvessel density. DCIS, ductal carcinoma in situ; EORTC, European Organization for Research and Treatment of Cancer and by Boehringer Mannheim.

### REFERENCES

Aihara M, Scardino PT, Truong LD, Wheeler TM, Goad JR, Yang G and Thompson TC (1995) The frequency of apoptosis correlates with the prognosis of Gleason Grade 3 adenocarcinoma of the prostate. *Cancer* **75**: 522–529

Bhargava V, Kell DL, van de Rijn M and Warnke RA (1994) Bcl-2 immunoreactivity in breast carcinoma correlates with hormone receptor Positivity. *Am J Pathol* **145**: 535–539

Blagosklonny MV, Schulte T, Nguyen P, Trepel J and Neckers LM (1996) Taxol-induced apoptosis and phosphorylation of Bcl-2 protein involves c-Raf-1 and represents a novel c-Raf-1 signal transduction pathway. *Cancer Res* **56**: 1851–1854

Bodis S, Siziopikou KP, Schnitt SJ, Harris JR and Fisher DE (1996) Extensive apoptosis in ductal carcinoma in situ of the breast. *Cancer* **77**: 1831–1835

Borner C (1996) Diminished cell proliferation associated with the death-protective activity of Bcl-2. *J Biol Chem* **271**: 12695–12698

Brady HJM, Gil-Gomez G, Kirberg J and Berns AJM (1996) Bax-α perturbs T cell development and affects cell cycle entry of T cells. *EMBO J* **15**: 6991–7001

Campos L, Rouault JP, Sabido O, Oriol P, Roubi N, Vasselon C, Archimbaud E, Magaud JP and Guyotat D (1993) High expression of bcl-2 protein in acute myeloid leukemia cells is associated with poor response to chemotherapy. *Blood* **81**: 3091–3096

Chresta CM, Masters JRW and Hickman JA (1996) Hypersensitivity of human testicular tumors to etoposide-induced apoptosis is associated with functional p53 and a high Bax:Bcl-2 ratio. *Cancer Res* **56**: 1834–1841

Clahsen PC, Van de Velde CJH, Duval C, Pallud C, Mandard A, Delobelle-Deroide A, van den Broek L, Sahmoud TM and van de Vijver MJ (1997) P53 expression and response to chemotherapy in premenopausal node-negative women with early breast cancer. *J Clin Oncol* (in press)

Donaldson KL, Goolsby GL, Kiener PA and Wahl AF (1994) Activation of p34cdc2 coincident with taxol-induced apoptosis. *Cell Growth Different* **5**: 1041–1050

Du M, Singh N, Husseuin A, Isaacson PG and Pan L (1996) Positive correlation between apoptotic and proliferative indices in gastrointestinal lymphomas of mucosa-associated lymphoid tissue (MALT). *J Pathol* **178**: 379–384

Evan GI, Wyllie AH, Gilbert CS, Littlewood TD, Land H, Brooks M, Waters CM, Penn LZ and Hancock DC (1992) Induction of apoptosis in fibroblasts by c-myc protein. *Cell* **69**: 119–128

Field SJ, Tsai FY, Kuo F, Zubiaga AM, Kaelin WG, Jr., Livingston DM, Orkin SH and Greenberg ME (1996) E2F-1 functions in mice to promote apoptosis and suppress proliferation. *Cell* **85**: 549–561

Gavrieli Y, Sherman Y and Ben-Sasson SA (1992) Identification of programmed cell death in situ via specific labeling of nuclear DNA fragmentation. *J Cell Biol* **119**: 493–501

Graeber TG, Osmanian C, Jacks T, Houseman DE, Koch CJ, Lowe SW and Giaccia AJ (1996) Hypoxia-mediated selection of cells with diminished apoptotic potential in solid tumours (see comments). *Nature* **379**: 88–91

Guan RJ, Moss SF, Arber N, Krajewski S, Reed JC and Holt PR (1996) 30 kDa phosphorylated form of Bcl-2 protein in human colon. *Oncogene* **12**: 2605–2609

Huang Y, Ray S, Reed JC, Ibrado AM, Tang C, Nawabi A and Bhalla K (1997) Estrogen increases intracellular p26bcl 2 to p21bax ratios and inhibits taxol induced apoptosis of human breast cancer MCF-7 cells. *Breast Cancer Res Treat* **42**: 73–81

Isacson C, Kessis TD, Hedrick L and Cho KR (1996) Both cell proliferation and apoptosis increase with lesion grade in cervical neoplasia but do not correlate with human papillomavirus type. *Cancer Res* **56**: 669–674

Joensuu H, Pylkkanen L and Toikkanen S (1994) Bcl-2 protein expression and long-term survival in breast cancer. *Am J Pathol* **145**: 1191–1198

Kerr JFR, Winterford CM and Harmon BV (1994) Apoptosis: Its significance in cancer and cancer therapy. *Cancer* **73**: 2013–2026

Ko LJ and Prives C (1996) p53: puzzle and paradigm. *Genes Dev* **10**: 1054–1072

Korsmeyer SJ (1995) Regulators of cell death. (Review). *Trends Genet* **11**: 101–105

Koshida Y, Saegusa M and Okayasu I (1996) Apoptosis, cell proliferation and expression of Bcl-2 and Bax in gastric carcinomas: immunohistochemical and clinocopathological study. *Br J Cancer* **75**: 367–373

Krajewski S, Blomqvist C, Franssila K, Krajewska M, Wasenius VM, Niskanen E, Nordling S and Reed JC (1995) Reduced expression of proapoptotic gene BAX is associated with poor response rates to combination chemotherapy and shorter survival in women with metastatic breast adenocarcinoma. *Cancer Res* **55**: 4471–4478

Kranenburg O, van der Eb AJ and Zantema A (1996) Cyclin D1 is an essential mediator of apoptotic neuronal cell death. *EMBO J* **15**: 46–54

Leek RD, Kaklamanis L, Pezzella F, Gatter KC and Harris AL (1994) Bcl-2 in normal human breast and carcinoma, association with oestrogen receptor-positive, epidermal growth factor receptor-negative tumours and in situ cancer. *Br J Cancer* **69**: 135–139

Lipponen PK and Aaltomaa S (1994) Apoptosis in bladder cancer as related to standard prognostic factors and prognosis. *J Pathol* **173**: 333–339

Lipponen P, Pietilainen T, Kosma VM, Aaltomaa S, Eskelinen M and Syrjanen K (1995) Apoptosis suppressing protein bcl-2 is expressed in well-differentiated breast carcinomas with favourable prognosis. *J Pathol* **177**: 49–55

Lu PJ, Lu QL, Rughetti A and Taylor-Papadimitriou J (1995) *bcl-2* overexpression inhibits cell death and promotes the morphogenesis, but not tumorigenesis of human mammary epithelial cells. *J Cell Biol* **129**: 1363–1378

Morgenbesser SD, Williams BO, Jacks T and DePinho RA (1994) p53-dependent apoptosis produced by Rb-deficiency in the developing mouse lens (see comments). *Nature* **371**: 72–74

Naik P, Karrim J and Hanahan D (1996) The rise and fall of apoptosis during multistage tumorigenesis: down-modulation contributes to tumor progression from angiogenic progenitors. *Genes Dev* **10**: 2105–2116

O'Reilly LA, Huang DCS and Strasser A (1996) The cell death inhibitor Bcl-2 and its homologues influence control of cell cycle entry. *EMBO J* **15**: 6979–6990

Pandey S and Wang E (1995) Cells en route to apoptosis are characterized by the upregulation of c-fos, c-myc, c-jun, cdc2, and RB phosphorylation, resembling events of early cell-cycle traverse. *J Cell Biochem* **58**: 135–150

Pietenpol JA, Papadopoulos N, Markowitz S, Wilson JKV, Kinzler KW and Vogelstein B (1994) Paradoxical inhibition of solid tumor cell growth by bcl-2. *Cancer Res* 3714–3717

Reed J (1994) Mini-review: cellular mechanisms of disease series. Bcl-2 and the regulation of programmed cell death. *J Cell Biol* **124**: 1–6

Sabourin JC, Martin A, Baruch J, Truc JB, Gompel A and Poitout P (1994) Bcl-2 expression in normal breast tissue during the menstrual cycle. *Int J Cancer* **59**: 1–6

Schneider HJ, Sampson SA, Cunningham D, Norman AR, Andreyev HJN, Tilsed JVT and Clarke PA (1997) Bcl-2 expression and response to chemotherapy in colorectal adenocarcinomas. *Br J Cancer* **75**: 427–431

Shi L, Nishioka WK, Th'ng J, Bradbury EM, Litchfield DW and Greenberg AH (1994) Premature p34cdc2 activation required for apoptosis (see comments). *Science* **263**: 1143–1145

Shimizu S, Eguchi Y, Kosaka H, Kamiike W, Matsuda H and Tsujimoto Y (1995*a*) Prevention of hypoxia-induced cell death by Bcl-2 and Bcl-$X_L$. *Nature* **374**: 811–813

© Cancer Research Campaign 1998

Shimizu T, O'Connor PM, Kohn KW and Pommier Y (1995b) Unscheduled activation of cyclin B1/Cdc2 kinase in human promyelocytic leukemia cell line HL60 cells undergoing apoptosis induced by DNA damage. *Cancer Res* **55**: 228–231

Shimizu S, Eguchi Y, Kamiike W, Waguri S, Uchiyama Y, Matsuda H and Tsujimoto Y (1996) Retardation of chemical hypoxia-induced necrotic cell death by Bcl-2 and ICE inhibitors: possible involvement of common mediators in apoptotic and necrotic signal transductions. *Oncogene* **12**: 2045–2050

Shoji Y, Saegusa M, Takano Y, Ohbu M and Okayasu I (1996) Correlation of apoptosis with tumour cell differentiation, progression, and HPV infection in cervical carcinoma. *J Clin Pathol* **49**: 134–138

Silvestrini R, Veneroni S, Daidone MG, Benini E, Boracchi P, Mezzetti M, Di Fronzo G, Rilke F and Veronesi U (1994) The bcl-2 protein: A prognostic indicator strongly related to p53 protein in lymph node-negative breast cancer patients. *J Natl Cancer Inst* **86**: 499–504

Silvestrini R, Benini E, Veneroni S, Daidone MG, Tomasic G, Squicciarini P and Salvadori B (1996) p53 and bcl-2 expression correlates with clinical outcome in a series of node-positive breast cancer patients. *J Clin Oncol* **14**: 1604–1610

Siziopikou KP, Prioleau JE, Harris JR and Schnitt SJ (1996) *bcl-2* expression in the spectrum of preinvasive breast lesions. *Cancer* **77**: 499–506

Sjogren S, Inganas M, Norberg T, Lindgren A, Nordgren H, Holmberg L and Bergh J (1996) The p53 gene in breast cancer: prognostic value of complementary DNA sequencing versus immunohistochemistry. *J Natl Cancer Inst* **88**: 173–182

Teixeira C, Reed JC and Pratt MAC (1995) Estrogen promotes chemotherapeutic drug resistance by a mechanism involving *Bcl-2* proto-oncogene expression in human breast cancer cells. *Cancer Res* **55**: 3902–3907

Theodorakis P, D'Sa-Eipper C, Subramanian T and Chinnadurai G (1996) Unmasking of a proliferation-restraining activity of the anti-apoptosis protein EBV BHRF1. *Oncogene* **12**: 1707–1713

van de Vijver MJ, Peterse JL and Mooi WJ (1988) Neu-protein overexpression in breast cancer. Association with comedo-type ductal carcinoma in situ and limited prognostic value in stage II breast cancer. *N Engl J Med* **319**: 1239–1245

van Slooten H, Clahsen PC, van Dierendonck JH, Duval C, Pallud C, Mandard A, Delobelle-Deroide A, Van de Velde CJH and van de Vijver MJ (1996) Expression of BCL-2 in node-negative breast cancer is associated with various prognostic factors, but does not predict response to one course of perioperative chemotherapy. *Br J Cancer* **74**: 78–85

Wang Q, Worland PJ, Clark JL, Carlson BA and Sausville EA (1995) Apoptosis in 7-hydroxystaurosporine-treated T lymphoblasts correlates with activation of cyclin-dependent kinases 1 and 2. *Cell Growth Different* **6**: 927–936

Wang J, Guo K, Wills KN and Walsh K (1997) Rb functions to inhibit apoptosis during myocyte differentiation. *Cancer Res* **57**: 351–354

Wang TT and Phang JM (1995) Effects of estrogen on apoptotic pathways in human breast cancer cell line MCF-7. *Cancer Res* **55**: 2487–2489

Williams BO, Remington L, Albert DM, Mukai S, Bronson RT and Jacks T (1994) Cooperative tumorigenic effects of germline mutations in Rb and p53. *Nature Genet* **7**: 480–484

Yao SL, Akhtar AJ, McKenna KA, Bedi GC, Sidransky D, Mabry M, Ravi R, Collector MI, Jones RJ, Sharkis SJ, Fuchs EJ and Bedi A (1996*a*) Selective radiosensitization of p53-deficient cells by caffeine-mediated activation of p34cdc2 kinase. *Nature Med* **2**: 1140–1143

Yao SL, McKenna KA, Sharkis SJ and Bedi A (1996*b*) Requirement of p34cdc2 kinase for apoptosis mediated by the Fas/APO-1 receptor and interleukin 1beta-converting enzyme-related proteases. *Cancer Res* **56**: 4551–4555

Yin XM, Oltvai ZN and Korsmeyer SJ (1995) Heterodimerization with Bax is required for Bcl-2 to repress cell death. *Curr Top Microbiol Immunol* **194**: 331–338

Zornig M and Evan GI (1996) Cell cycle: on target with Myc. *Current Biol* **6**: 1553–1556

© Cancer Research Campaign 1998